# Loneliness modulates the neural dynamics of language processing in healthy older adults: evidence from event-related potentials

**DOI:** 10.1093/scan/nsaf030

**Published:** 2025-04-10

**Authors:** Bing Li, Chih-Mao Huang, Ya-Yi Wang, Qiduo Lin, Hsu-Wen Huang

**Affiliations:** National Center for Geriatrics and Welfare Research, National Health Research Institutes, Miaoli 350401, Taiwan; Department of Biological Science and Technology, National Yang Ming Chiao Tung University, Hsinchu 300093, Taiwan; Center for Intelligent Drug Systems and Smart Bio-devices (IDS2B), National Yang Ming Chiao Tung University, Hsinchu 300093, Taiwan; Department of Speech Language Pathology and Audiology, Chung Shan Medical University, Taichung 40201, Taiwan; Institute of Linguistics, Academia Sinica, Taipei 115201, Taiwan; National Center for Geriatrics and Welfare Research, National Health Research Institutes, Miaoli 350401, Taiwan; Department of Linguistics and Translation, City University of Hong Kong, Hong Kong, China; National Center for Geriatrics and Welfare Research, National Health Research Institutes, Miaoli 350401, Taiwan; Center for Intelligent Drug Systems and Smart Bio-devices (IDS2B), National Yang Ming Chiao Tung University, Hsinchu 300093, Taiwan

**Keywords:** aging, loneliness, semantic processing, event-related potential, N400

## Abstract

Loneliness, a distressing emotional response to perceived deficiencies in social interactions, has seen a marked increase in prevalence since the COVID-19 pandemic. While previous research has linked loneliness in older adults to affective disorders and cognitive decline, its impact on language comprehension—a crucial aspect of social interaction—remains underexplored. This study addresses this gap by examining the effects of loneliness on semantic retrieval in healthy older adults. Using event-related potentials, we measured participants’ neural responses as they verified category membership across three conditions: high typicality, low typicality, and category violations. We found that loneliness was negatively correlated with an N400 amplitude reduction for low-typicality items compared to category violations. Moreover, individuals who reported a high level of loneliness exhibited attenuated and delayed N400 effects within more restricted time windows compared to their less lonely counterparts. These results indicate that loneliness impairs semantic memory retrieval in older adults, potentially compromising language comprehension and further exacerbating social isolation. This research highlights the detrimental impact of loneliness on linguistic abilities, which may contribute to a vicious cycle of increasing social isolation and deepening loneliness.

## Introduction

Humans are naturally social beings, and language abilities, including comprehension and production, are vital for sustaining social interactions. Loneliness, a subjective and distressing emotional state that arises when the desired level of social interaction is not satisfied (Weiss 1973, Perlman and Peplau 1981), is a significant concern among the elderly due to its extensive prevalence and detrimental impact on overall well-being (Boss et al. 2015, Holt-Lunstad and Perissinotto 2023).

Research has shown that loneliness in late adulthood is associated with physical health issues (Cacioppo and Hawkley 2003) and a higher than average mortality rate (Stessman et al. 2014, Rico-Uribe et al. 2018, Ward et al. 2021). Loneliness also significantly impacts mental health, exacerbating conditions such as anxiety (Santini et al. 2020), late-life depression (Sin et al. 2021), and cognitive decline (Lara et al. 2019). A meta-analysis by Donovan et al. (2017) highlighted that loneliness is a major risk factor for cognitive decline and the progression of Alzheimer’s disease and other types of dementia. Moreover, loneliness contributes to structural and functional changes in the brain (as reviewed by Lam et al. 2021). Cognitively, loneliness in older adults has been linked to impaired working memory (Sin et al. 2021), decreased verbal fluency, and worsened memory performance (Shankar et al. 2013). These findings suggest that loneliness has significant, multifaceted impacts on the aging population. However, there is a notable lack of research addressing how loneliness influences language processing, leaving a critical gap in our understanding of this issue (Zhou et al. 2022), despite the fact that the affected cognitive abilities are integral to language processing, and, more importantly, language abilities are fundamental to maintaining self-efficacy in late adult life.

Studies examining age-related changes in language functioning have reported mixed results for different aspects of language ability. For instance, word-level knowledge remains relatively stable and preserved in the aging population (Verhaeghen 2003, Huang et al. 2012, Stine-Morrow and McCall 2022), as assessed by word associations (Burke and Peters 1986) and semantic priming (Laver and Burke 1993). However, the ability to use context and integrate information in a timely manner to achieve sentence comprehension is significantly delayed in older adults, and age-related alterations in language-processing neural mechanisms have been observed (Kutas and Iragui 1998, Wlotko and Federmeier 2012, Wlotko et al. 2012, Payne and Federmeier 2018). Older adults are less likely to engage in anticipatory and predictive processing and rely more on bottom-up features due to less efficient activation of long-term semantic memory (Huang et al. 2012, Wlotko et al. 2012, Payne and Federmeier 2018). More importantly, decline in cognitive ability varies greatly among the elderly (for a review, see Huang and Huang 2019), which suggests that there is considerable variance in language processing capacity among older adults. Indeed, research has shown that variances in working memory capacity (Payne and Stine-Morrow 2012, Payne et al. 2014), reading experience (Payne et al. 2012, 2014), and verbal fluency (Federmeier et al. 2010) contribute to the individual differences in language comprehension performance and strategies employed by older adults. A recent behavioral study demonstrated that loneliness has adverse effects on word reading (Zhou et al. 2022), although the underlying neural mechanisms for this are still unclear (Li and Huang 2024).

The aim of this study was to understand whether, in what aspects, and to what extent loneliness affects language processing in healthy older adults. Given the fact that aging has been linked to reduced effectiveness in preparing for upcoming words and building an integrated sentence-level representation (Huang et al. 2012, Wlotko and Federmeier 2012, Wlotko et al. 2012), it would be difficult to assess the impact of loneliness with a sentence processing task. Therefore, this study used simpler language units with a category cue and target word pair in a category membership verification task. This type of task is commonly used to examine semantic memory organization and meaning retrieval, which are core mechanisms in language processing (Heinze et al. 1998, Riley et al. 2018). In category verification tasks, the processing and verification of target words is facilitated by the activation of semantic associations primed and activated by category cues, to a degree that depends on how the targets are associated with the category cue. Ample evidence has verified that typical category exemplars that are the most frequently generated response to the category cues (e.g. “sparrow” following “a bird”) have shorter reaction times (e.g. Rosch 1973) and greater accuracy (Fujihara et al. 1998) than atypical exemplars (e.g. “penguin” following “a bird”), the so called “typicality effect.”

In addition to traditional behavioral measurements such as accuracy and reaction times, electroencephalogram (EEG) recordings provide event-related potential (ERP) data that enable real-time examination of the neural dynamics underlying semantic retrieval and integration. ERPs offer a direct, temporally precise, multidimensional view of brain activity, including functionally specific neural markers for aspects of perception, attention, memory, and language (Fabiani et al. 2007), making them particularly well suited to explorations of how and when the brain extracts meaning from linguistic input. For example, the primary brain potential component used to index the cognitive process of meaning access and integration is the N400, a negative-going component approximately in the time window of 250–550 ms and peaking at 400 ms after stimulus onset (Kutas and Federmeier 2011). A more negative N400 indicates more effortful meaning access/integration due to the low utility of the preceding context in facilitating the processing of the targets, such as when they are semantically incongruent, or building an expectation for an upcoming word or sentence completion that does not appear to match the stimulus (Federmeier 2021). When the category verification task is combined with the EEG technique, ERP data consistently show that typical exemplars elicit less negative N400s than atypical exemplars and non-category targets (Stuss et al. 1988, Fujihara et al. 1998, Heinze et al. 1998). ERP research has also demonstrated that healthy older adults show typicality effects on the N400 with similar patterns to young participants (Federmeier et al. 2010). These findings suggest that older adults as a group are able to use the category cue to predict and activate target words when the task is simple.

To gain a comprehensive understanding of the impact of loneliness on the use of context in meaning retrieval among the elderly and its neural mechanisms, we combined a category verification task with ERP measures. If loneliness hinders predictive processing in word activation (Zhou et al. 2022), there should be individual differences in N400 effects driven by participants’ loneliness levels.

## Methods

### Participants

Fifty community-dwelling elderly individuals were recruited. Seven participants were excluded from the statistical analyses due to the high artifact rejection rate in ERP preprocessing, which results in the final sample size of 43. Table 1 presents demographic information of 43 participants. All of the participants were native Chinese speakers and were right-handed as assessed by the Edinburgh Handedness Inventory (Oldfield 1971). They were screened for cognitive and psychological conditions using the Taiwanese version of the Montreal Cognitive Assessment (MoCA-T; Tsai et al. 2012) and Geriatric Depression Scale (GDS; Yesavage et al., 1982), respectively. All participants passed the screening (no worse than “mildly impaired,” MoCA > 18; “mildly depressed,” GDS < 19). They self-reported normal general health and normal or corrected-to-normal eyesight.

**Table 1.** Participant characteristics.

Demographics information	
Gender (Female/Male)	30/13
Marital status (Married/Non-married (“Non-married” includes divorced, single, and widowed.))	29/14
	Mean (s.d.)
Age (years)	66.0 (4.9)
Years of Education	14.3 (3.0)
**Cognitive and psychological assessments**	
MoCA	24.7 (2.77)
GDS	6.2 (3.93)
UCLA-LS	38.2 (7.82)
CVF	
CVF_Average	17.2 (2.32)
CVF_Animal	17.60 (4.28)
CVF_Fruit	15.09 (2.74)
CVF_Color	15.79 (4.12)
CVF_Taiwan	20.40 (3.67)

Note. MoCA= Montreal Cognitive Assessment; GDS= Geriatric Depression Scale; UCLA-LS= the UCLA Loneliness Scale; CVF= Category Verbal Fluency. CVF_Average is the averaged verbal fluency score across the four sub-categories.

Perceived loneliness was measured using the 20-item University of California Loneliness Scale (UCLA-LS, Russell 1996, Russell et al. 1980). This scale uses a 4-point Likert scale (1 = never to 4 = always), with higher total scores indicating a higher level of perceived loneliness. A category verbal fluency test (CVF, Chung et al. 2007) was administered to assess the participants’ verbal ability. For this test, the participants were asked to generate as many examples as possible in 60 s from the categories of animals, fruits, colors, and names of counties/towns in Taiwan, in separate sessions. The score was the number of correct and non-repeated words. All the participants provided informed consent before data collection and were compensated for their time with cash. This study was approved by the Institutional Review Board of Academia Sinica, Taiwan.

### Materials and procedures

The stimuli consisted of 90 category cues, each of which was paired with three target words—high typicality (HT), low typicality (LT), and category violation (VIO)—resulting in a set of 270 category–target pairs. These target words were matched for the part of speech (most were nouns), and word length (most were two-character words). The full list of category cues, HT and LT exemplars were adapted and modified from the existing norms (Yoon et al. 2004, Li et al. 2021). The typicality and familiarity of the target words were then assessed by native speakers of Taiwanese Mandarin in two norming studies. In the typicality norming study, 60 students (30 females, mean age 23 years, range 20–28) participated and were compensated with cash. A total of 288 category–target pairs were divided into three lists; within each, 96 category-target pairs were included. Each participant completed one list. Participants used a 1–7 rating scale to quantify the typicality of target words and their respective categories with “7” indicating the most typical and “1” indicating the least typical. All the target words were rated for the familiarity. In this norming, 20 students (10 females, mean age 22 years, range 20–26) participated and were compensated with cash. Subjects were instructed to indicate the subjective level of familiarity with the word based on their daily language experience, with a 1–7 scale (7 indicating very familiar and 1 indicating extremely unfamiliar). No participant took part in two norming studies. All of the participants provided informed consent before data collection. The typicality norming was designed to verify the manipulation of the task conditions. The familiarity norming was designed to ensure that all of the target words were familiar words to avoid a confounding effect on N400, the component of interest for our analysis.

The final set of stimuli, 270 category–target pairs, was divided into three lists, such that each category cue was paired with one target within a list. Each participant completed one list of 90 trials, with 30 trials in each condition. The sample materials and mean values of typicality and familiarity for the target words are shown in Table 2.

**Table 2.** Sample materials and ratings.

	Category	HT	LT	VIO
Sample materials	一種燃料 Fuel	汽油 Petrol	樹枝 Tree branches	果凍 Jello
	一種家用電器 Home appliance	電視 TV	熨斗 Iron	貢丸 Meatball
Mean (s.d.)	Typicality	6.60 (0.28)	5.30 (0.80)	1.30 (0.53)
	Familiarity	5.32 (0.73)	4.98 (0.66)	5.86 (0.69)

For the ERP sessions, the participants viewed the stimuli while sitting in a soundproofed chamber. They were told that two words would appear in succession for each trial; the first word would be a short category cue, and the second would be a target word. They were instructed to silently generate in their mind category exemplars when the category names were presented. Then, when a prompt page was shown, they were asked to indicate their category membership judgment after viewing the target word by pressing “1” on a response pad if the target word was the exact word they had generated as the category exemplar, “2” if the target was not generated but was acknowledged as a category member, or “3” if the target word was to be rejected for category membership.

For each trial, a fixation cross appeared for 500 ms, followed by a jittered interval of 750–1000 ms. A category name was then presented for 2150 ms, followed by another jittered interval of 750–1000 ms before the target word was shown for 1000 ms. Then, a 500 ms blank frame was used to mask the target word. A question mark was presented as the prompt page until the participant responded or for a maximum of 2000 ms (delayed response). The participants thus had ample time to activate categorical semantic associations primed by the category cue and then to get ready to register their verification decision after reading the target words. There were three blocks of trials, with 30 trials per block. Between blocks, the participants took a short break. The total EEG recording time was 15 min. Before the critical trials, a practice session consisting of nine trials was conducted to familiarize the participants with the task procedure and the testing environment.

EEGs were recorded using a 32-channel cap with Ag/AgCl electrodes (QuikCap). Signals were amplified using a SYNAMPS2 device (Neuroscan, Inc.) and bandpass filtered between 0.1 and 100 Hz and digitized at 500 Hz. Data were referenced to the average of the left and right mastoids. Two virtual channels, VEOG and HEOG, were created to detect eye movement artifacts. The VEOG was computed as the numerical difference between the upper and lower electrodes on the left eye to detect blinks. The HEOG was computed as the amplitude difference between electrodes placed on the outer corners of the eyes to detect horizontal eye movements. ERPs were computed from 200 ms prestimulus baseline to 900 ms poststimulus onset for the target words. Trials containing horizontal eye movements, blinks, excessive body movements or other recording artifacts were excluded from further analysis. A digital bandpass filter of 0.1–30 Hz was applied to the ERPs before measurements. All artifact-free trials were averaged by condition. Approximately 11.81% of the trials were lost due to artifacts, and seven participants were excluded from the analysis due to an insufficient number of remaining trials. Statistical analyses were performed on mean amplitudes in the N400 window, and all reported *P*-values are after Epsilon correction (Greenhouse–Geisser).

## Results

### Behavioral results

The behavioral results include accuracy rates for category membership verification and the expected-response rates for the three conditions. Accuracy rates are the proportions of items correctly verified as members of a given category by pressing “1” or “2” for HT, “1” or “2” for LT, and only “3” for VIO. Expected-response rates are the proportions of items in the HT condition with a response of “1,” LT with “2,” and VIO with “3.” Reaction times are not included in the analysis due to the delayed-response design of the experiment.

**Figure 1.**
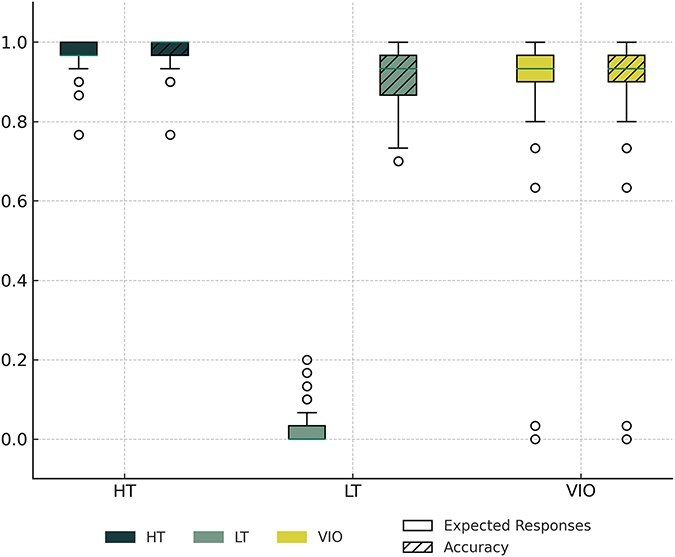
Expected-response rates and accuracy. Accuracy rates, i.e. the proportion of items correctly verified as members of a category, were high. Expected-response rates are the proportions of items that were responded to as follows: for high typicality (HT), the proportion of responses of “1, I thought of this exact word”; for low typicality (LT), the proportion of responses of “2, I did not think of this word, but I agree it belongs to this category”; and for category violation (VIO), the proportion of responses of “3, I don’t think the target belongs to this category.”.

As shown in the left panel of Fig. 1, the overall accuracy rates for category membership verification in the three conditions were high: 97.44% for HT, 90.62% for LT, and 87.98% for VIO. LTs were verified by their significantly lower accuracy rates than HTs (*t* = 1.99, *P* < .001). As shown in the right panel of Fig. 1, the expected-response rate for LT was far lower than for the other conditions, especially HT (96.82% of HT responses were “1,” 2.79% of LT responses were “2”). On average, the participants only missed 1.95 of the 90 trials (s.d. = 3.55, range: 0–18), indicating adequate response rates.

### ERPs

**Figure 2.**
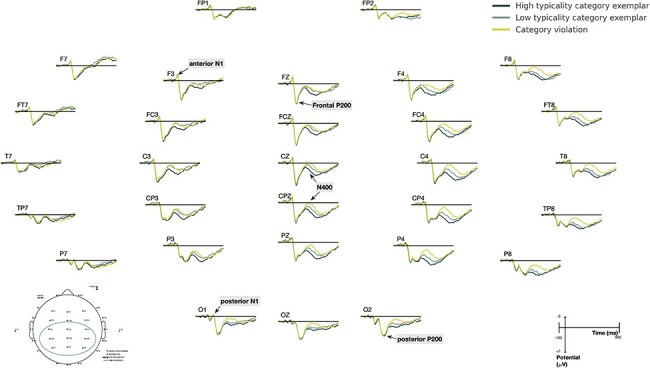
Grand average ERPs for all 43 participants. Early visual evoked components: the posterior N1 (190 ms) and P200 (280 ms), as well as the anterior N1 (130 ms) and frontal P200 (200 ms), which were followed by a typical N400 (300–550 ms) in all three conditions are indicated with arrows.

The grand average ERPs for all 43 participants are shown in Fig. 2. As illustrated, all three conditions elicited typical visual evoked potentials, including the posterior N1 (190 ms), and P200 (280 ms), as well as the anterior N1 (130 ms) and frontal P200 (200 ms). Following these early components, a negative N400 was observed in all of the conditions.

### Condition effects on N400 for all participants

The N400 component is widely distributed across the scalp in the time window of 300–550 ms. The observed waveforms suggested a right-lateralized centro-parietal enhancement of amplitudes and effects, which is consistent with the descriptions of N400 in previous studies (for a review, see Kutas and Federmeier 2011). The statistical analysis of N400 amplitudes and effects was finalized as the region of the nine centroparietal electrodes of C3, CZ, C4, CP3, CPZ, CP4, P3, PZ, and P4. A 3 (condition) × 9 (electrode) repeated measures ANOVA showed a condition main effect (*F*(1.99, 83.460) = 25.96, *P* < .001). Post-hoc tests revealed that HT elicited the least negative N400 (*P’*s < .001) and VIO the most negative (compared to HT, *p* < .001; compared to LT, *p* < .01; Bonferroni-corrected). The N400 responses of LT were intermediate between HT and VIO. The graded N400 effects are in line with previous findings (Federmeier et al. 2010).

### Correlations between N400 effects and loneliness level

To examine whether verbal abilities and level of loneliness modulated the participants’ semantic processing, the correlations of N400 effects with CVF scores and loneliness level were analyzed separately. An N400 effect was defined as the difference between the mean amplitudes of the conditions in the time window of 300–550 ms. The three N400 effects were the typicality effect (TypE, the N400 mean amplitude difference between HT and LT), the HT category effect (HTCatE, between HT and VIO), and the LT category effect (LTCatE, between LT and VIO).

**Figure 3.**
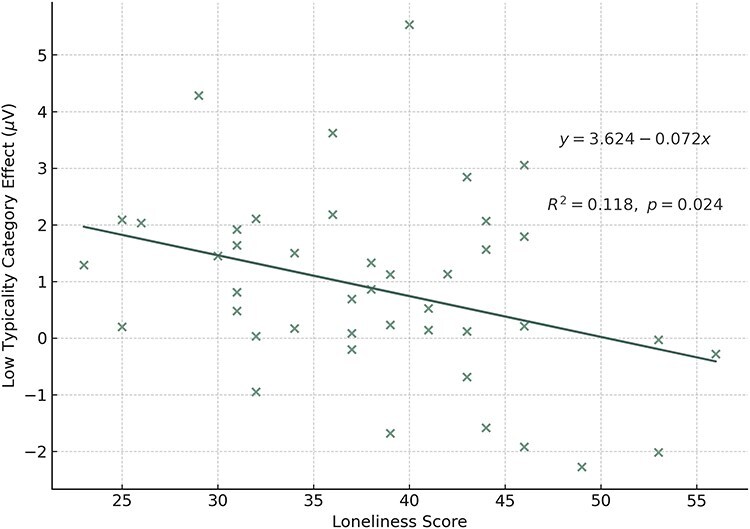
The correlation between low-typicality category effect (LTCatE) and loneliness. The LTCatE is the N400 amplitude difference between LT and VIO targets. The LTCatE was negatively correlated with loneliness, the higher the loneliness score, the smaller the N400 difference between the LT and the VIO.

No significant correlations were identified between the N400 effects and CVF scores (whether averages or category scores), nor the CVF and Loneliness. Importantly, loneliness was moderately and negatively correlated with LTCatE (*r* = − 0.343, *P* < .05, Fig. 3) after controlled for CVF. Subjects with higher loneliness scores showed smaller N400 differences between LT and VIO.

### Comparison between high and low loneliness groups

To further examine the effect of loneliness on categorical semantic information processing, data from the 16 participants with the highest and 16 with the lowest scores on the UCLA-LS were extracted to form a high loneliness group (HLG) and a low loneliness group (LLG), respectively. The LLG had a significantly lower mean loneliness score (Mean = 30.13, s.d. = 3.65) than the HLG (Mean = 46.19, s.d. = 4.37; *t*(30) = − 11.29, *P* < .001). The extreme group approach was used to ensure that the groups represent the extremes of the loneliness spectrum (Preacher 2015), allowing for clearer interpretation of any observed difference in semantic processing.

The behavioral results revealed no significant differences between the LLG and HLG. Specifically, there were no significant differences in the proportions of “1” responses on the HT, the accuracy of confirming HT and LT category members, the accuracy of rejecting VIO as a category member (“3” responses), or the response rates (all *P’s* > .05). The “2” response was used infrequently in both groups.

**Figure 4.**
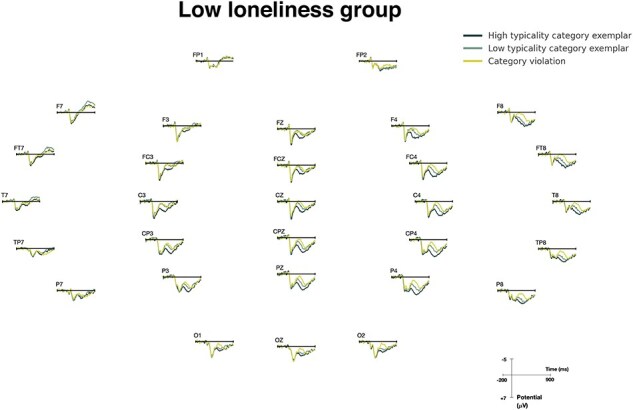
Grand average ERPs of the LLG.

**Figure 5.**
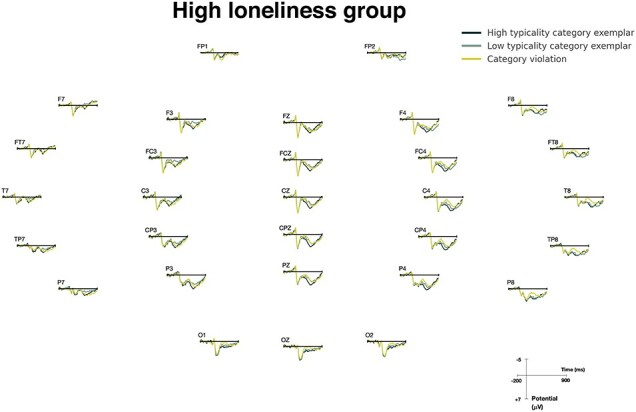
Grand average ERPs of the HLG. Compared to the LLG (Fig. 4), the HLG showed overall reduced differences between conditions.

**Figure 6.**
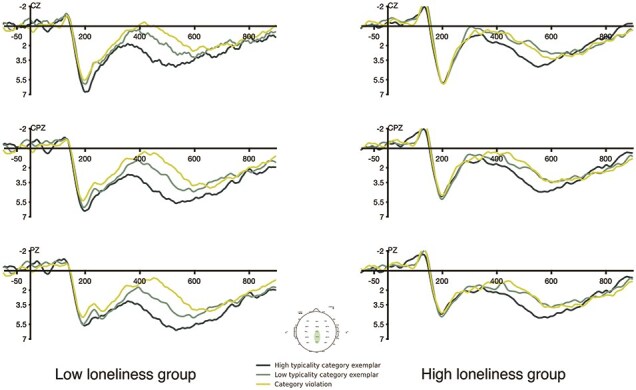
ERP waveforms at CZ, CPZ, and PZ for the LLG and HLG. N400 responses were attenuated in the HLG compared to the LLG. In particular, the difference between LT and VIO was almost absent in the HLG.

The ERP results of the two groups are shown in Fig. 4 (LLG) and Fig. 5 (HLG). A repeated measures ANOVA was conducted on the N400 mean amplitudes, with three conditions and nine electrodes as within-subject factors and loneliness level as the between-subjects factor. The analysis revealed an expected significant main effect of condition (*F*(1.96, 58.67) = 26.03, *P* < .001) and a significant condition × loneliness-level interaction (*F*(1.96, 58.67) = 4.103, *P* < .05). Post-hoc comparisons were conducted to further investigate this interaction effect (Bonferroni corrections applied). For the HLG, the only significant N400 difference was for HTCatE, which was marginally significant after correction (*P* = .076). For the LLG, the TypE was significant (*P* < .01) and so was the HTCatE (*P* < .001). ERP waveforms are plotted at three representative electrodes (CZ, CPZ, and PZ) for the two groups in Fig. 6.

### Successive time window analysis of N400

The statistical analysis revealed attenuated overall N400 effects in the HLG. Further inspection of the waveforms suggested time-course differences. To explore these temporal differences between two groups, we segmented the N400 time window into successive 50 ms time periods from 300 to 550 ms. For each time window, the N400 mean amplitude was subjected to a repeated measures ANOVA on a 3 (condition) × 9 (electrode) design, with loneliness level as the between-subjects factor.

Table 3 reports the corrected-significance levels of the condition main effects, the condition × loneliness level interaction, and the three N400 effects (HTCatE, LTCatE, and TypE) of the two groups. HTCatE was significant for most of the successive windows for the LLG, but only significant in the last consecutive 100 ms in the HLG. A similar pattern was found for TypE. These results coincided with the waveforms shown in Fig. 4b, confirming that the LLG demonstrated earlier onset of N400 effects and more significant differences for the 50 ms windows. Overall, the HLG showed significant HTCatE with a delayed onset in the 450–500 ms window compared to the LLG. LTCatE was absent in the HLG for all time windows. Additionally, the TypE was only significant for the 500–550 ms window. These findings suggest that the N400 effects were more delayed and attenuated in the HLG than the LLG.

**Table 3.** Successive 50 ms time-window analysis of N400.

		Condition × Loneliness	HTCatE		LTCatE		TypE	
	Condition		LLG	HLG	LLG	HLG	LLG	HLG
300–350	*							
350–400	** *		*				*	
400–450	** *	*	** *				**	
450–500	** *	*	** *	*			** *	
500–550	** *	*	** *	**	**		**	**

Note*. P *< .05, *; *P* < .01, **; *P* < .001, ***.

## Discussion

This study investigated how perceived loneliness influenced neural responses and brain dynamics during semantic processing in a category membership verification task. Using ERP data from 43 healthy elderly participants, we found a negative correlation between the low-typicality category effect and UCLA loneliness score, which motivated a group-level analysis. Participants in the low- and high-loneliness groups showed no differences in verifying category memberships in their behavioral responses. In contrast, the N400 responses showed a significant loneliness level interaction. A smaller N400 amplitude reduction for low-typicality exemplars compared to category violations in the HLG suggested more limited activation and utilization of context information from the category cue, such that semantic facilitation was restricted to the best-fitting targets, i.e. the high-typicality exemplars. The N400 process was then further characterized using successive window analysis, and the N400 effects of high loneliness individuals were found to be attenuated and with delayed onset in multiple 50 ms windows.

In general, the participants in this experiment accomplished the verification task with high accuracy. This is in agreement with previous findings on preserved lexical abilities in healthy older adults (Verhaeghen 2003, Stine-Morrow and McCall 2022), especially considering the task was simple. Interestingly, even though the current study used a tri-choice design for registering subjects’ behavioral responses, the participants still demonstrated polarized response patterns. They responded to category exemplars (HT and LT) predominantly with “1” (“I thought of the exact word, and it is a category member”) and sporadically with “2” (“I did not think of the exact word, but I agree it is a category member”), and responded to category violations with “3” (the word is not a category member). This pattern of behavior may be due to the use of the delayed response paradigm, which requires participants to retain the words they generated when reading the category cue, make comparisons when the target words appear, and then register their responses. Therefore, to reduce cognitive demand, the participants may not have differentiated their behavioral responses to target words that were category members, regardless of whether they had thought of the word or not. Thus, the behavioral results do not show whether the participants were using the category cue to predict the word. In contrast, the ERP results demonstrated that as a group the participants elicited typical N400 responses in the central-parietal regions and graded N400 responses between conditions, indicating their preserved semantic processing. The graded N400 responses, especially the intermediate N400 responses for LT, also suggest that the participants used the category cue to activate the target words.

The correlational analyses of the verbal fluency and N400 effects did not reach statistical significance. This outcome is not surprising, given that the verbal fluency task did not depend solely on word knowledge. It also entailed self-initiated memory retrieval, information updating, and inhibition processes. Previous research has demonstrated that verbal fluency has an impact on the later processing stages, such as meaning revision, rather than on the earlier semantic activation (Federmeier et al. 2010). Strikingly, loneliness hindered the activation and retrieval of related-category semantic associations, as indicated by the negative correlation between LT-VIO N400 effects and loneliness level. This finding suggests that distinctions between remotely related and unrelated words become muddled as loneliness increases.

A group comparison of ERPs further revealed the impact of loneliness on semantic processing when comparing individuals with extreme loneliness scores. Participants with low levels of perceived loneliness demonstrated significant effects of typicality, HTCatE, and LTCatE, indicating their intact semantic network organization and normal semantic functioning. Those with high loneliness levels, in contrast, only showed a marginal effect of HTCatE, while typicality and LTCatE were absent. Successive window analysis with chunked observations in the average time window along the processing time course showed that high-loneliness individuals exhibited a delayed onset and more restricted period of the N400 effects, with a late HTCatE and a rapidly disappearing typicality effect only at the end of the N400 component. These results suggest less efficient activation of associative information in the category context and/or an alteration in the organization of semantic memory for high-loneliness older adults compared to low-loneliness older adults. The diminished typicality effect indicates that items within a category lose their distinction, while the difficulty in distinguishing between LT and violations highlights impairments in finer-grained semantic discrimination.

Moreover, the attenuated N400 observed among high-loneliness individuals may not solely reflect reduced predictive activation. An alternative interpretation involves deficits in attentional control and working memory, which are critical for integrating contextual information and allocating cognitive resources effectively during semantic processing. Loneliness has been associated with diminished executive functioning and impaired resource allocation (Cacioppo and Hawkley 2009, Sin et al. 2021), potentially limiting the ability to maintain focus on task-relevant information or suppress irrelevant stimuli. Such resource allocation deficits may further exacerbate difficulties in activating and retrieving semantic associations, especially for less typical or weakly related concepts, contributing to the delayed and restricted N400 effects observed. These findings align with previous research showing that gradients of relatedness became dysfunctional among patients exhibiting early stages of dementia (Grober et al. 1985) and probable Alzheimer’s disease (Nebes 1989). Future studies could investigate the specific contributions of attentional control and working memory in mediating the effects of loneliness on semantic processing, as this may provide a more comprehensive understanding of the observed impairments.

These cognitive vulnerabilities provide important insight into the broader implications of loneliness, which has increasingly been linked to dementia and Alzheimer’s disease (e.g. Sundström et al. 2020). The physiological results observed in the current study shed light on how loneliness can corrode the rapid cognitive mechanisms involved in making sense of the world. Considering the unique social nature of language, an impairment of linguistic functioning can be a direct threat to interpersonal communication and social interaction, both of which are vital for maintaining a functional independent daily life. Category semantic processing reflects the basic implementation of semantic memory, and the current study found that the affected semantic sensitivity lies in the differences between a legitimate atypical category exemplar and a non-member. Instead of being less distinctive within a category, a reduced LT–VIO difference puts one on the edge of being right or wrong for a category judgment. This can lead to general misunderstandings and inefficiencies in verbal exchanges with others, which in turn can exacerbate frustration with negative social interactions and decrease the willingness to take social risks to alleviate loneliness, potentially leading to defensiveness, hostility, and withdrawal. Insufficient social interactions may also limit opportunities to maintain proper language functions. Thus, the effect of loneliness on basic level semantic processing may be a mediator of the reciprocal relationship between the progression of loneliness and changes in individuals’ social behavioral patterns.

When examining the negative impact of loneliness on quality of life in late adulthood, it is essential to consider the role of fully functional language processing ability. Deteriorating language processing abilities have the potential to exacerbate loneliness, creating a harmful feedback loop that leads to cognitive decline and the development of more severe cognitive impairments. These impairments can in turn lead to further language processing declines, thereby escalating social isolation by obstructing effective communication.

The results of this study have both theoretical and practical relevance. Loneliness in the elderly has been shown to be effectively alleviated through various interventions, such as increased visits, implementing communication technology, and social activities in nursing homes (e.g. Chen and Schulz 2016; for a review, see Cohen-Mansfield and Perach 2015). As loneliness has now been linked to declines in functional semantic processing, attention should be given to both of these factors, with more sensitive and functional assessments and interventions for language processing taken into consideration.

## Conclusion

This study characterized how loneliness affects basic categorical semantic processing in healthy older adults by using high temporal resolution electrophysiological data. Loneliness was found to attenuate N400 neural responses and to compromise efficacy in activating and retrieving semantic information to facilitate the processing of low-typicality exemplars from category contexts. Individuals with high levels of loneliness had a delayed onset and diminished N400 effects compared with those who reported feeling less lonely and were more satisfied with the quality and quantity of their social interactions. These results expand our understanding of the impact of loneliness on the cognitive functioning of healthy older adults in terms of category semantic processing and comprehension.
